# The Development of a Reverberation Chamber for the Assessment of Biological Effects of Electromagnetic Energy Absorption in Mice

**DOI:** 10.1002/bem.22539

**Published:** 2025-01-15

**Authors:** Steve Iskra, Robert L. McIntosh, Raymond J. McKenzie, John V. Frankland, Chao Deng, Emma Sylvester, Andrew W. Wood, Rodney J. Croft

**Affiliations:** ^1^ 6G Research and Innovation Lab Swinburne University of Technology Hawthorn Australia; ^2^ Telstra Limited Melbourne Australia; ^3^ Australian Mobile Telecommunications Association (AMTA) Melbourne Victoria Australia; ^4^ School of Medical, Indigenous and Health Sciences University of Wollongong Wollongong Australia; ^5^ Bioelectromagnetics Laboratory University of Wollongong Wollongong Australia; ^6^ School of Health Sciences Swinburne University of Technology Hawthorn Australia; ^7^ School of Psychology University of Wollongong Wollongong Australia

**Keywords:** computational modeling, reverberation chamber, SAR validation, uncertainty

## Abstract

In this paper, we present the design, RF‐EMF performance, and a comprehensive uncertainty analysis of the reverberation chamber (RC) exposure systems that have been developed for the use of researchers at the University of Wollongong Bioelectromagnetics Laboratory, Australia, for the purpose of investigating the biological effects of RF‐EMF in rodents. Initial studies, at 1950 MHz, have focused on investigating thermophysiological effects of RF exposure, and replication studies related to RF‐EMF exposure and progression of Alzheimer's disease (AD) in mice predisposed to AD. The RC exposure system was chosen as it allows relatively unconstrained movement of animals during exposures which can have the beneficial effect of minimizing stress‐related, non‐RF‐induced biological and behavioral changes in the animals. The performance of the RCs was evaluated in terms of the uniformity of the Whole‐Body Average‐Specific Absorption Rate (WBA‐SAR) in mice for a given RF input power level. The expanded uncertainty in WBA‐SAR estimates was found to be 3.89 dB. Validation of WBA‐SAR estimates based on a selected number of temperature measurements in phantom mice found that the maximum ratio of the temperature‐derived WBA‐SAR to the computed WBA‐SAR was 1.1 dB, suggesting that actual WBA‐SAR is likely to be well within the expanded uncertainties.

## Summary

- A full description of the design and development of two reverberation chambers (RC) is presented.
- A comprehensive uncertainty analysis of the RCs is presented. A good understanding in the potential range of given Whole‐Body Average‐Specific Absorption Rate (WBA‐SAR) values is essential when assessing possible biological effects due to the radiofrequency electromagnetic fields (RF‐EMF).
- Validation of WBA‐SAR estimates is performed using temperature measurements based on the “lumped‐heat‐capacity” method.

## 1 Introduction

At the University of Wollongong's Bioelectromagnetics Laboratory (UoWBL), Wollongong, Australia, research is underway to consider whether radiofrequency (RF) electromagnetic field (EMF) exposure results in biological effects in mice and to ascertain an improved understanding of their thermophysiological response due to the RF‐EMF. This paper will focus on the information gained from the development of two reverberation chambers (RC), used as RF‐EMF exposure systems for the studies at UoWBL.

UoWBL is undertaking a partial replication of research undertaken in Korea by Jeong et al. (2015), Son et al. (2016), Son et al. (2018), and Jeong et al. (2020), who reported that RF‐EMF exposure of mice within an RC resulted in either non‐harmful or beneficial changes to cognition and Alzheimer's disease (AD) pathology in mice predisposed to AD when compared with a sham control group placed in a separate RC. For the exposed group of mice in the experiments, the RF‐EMF was generated at a frequency of 1950 MHz (continuous wave [CW]), and the power level set to provide a Whole‐Body Average‐Specific Absorption Rate (WBA‐SAR) of 5 W/kg. At UoWBL, the studies will similarly include the use of two RCs, either of which can be assigned for the RF‐EMF exposed group and the other for the sham‐exposed group (for each loading of mice, the respective chamber is chosen randomly by a software‐initiated blinding process). The software was developed to control exposure levels in the chambers, monitor the state of exposure during experiments, and facilitate double‐blinding. This includes not displaying which of the two RCs has RF‐EMF exposure. Each RC will have eight plastic cages, with four mice placed in each cage.

A separate study at UoWBL, which used thermal sensors implanted within the intraperitoneal cavity of wild‐type mice, has provided a good understanding of the thermophysiological response of mice to RF‐EMF and is discussed in Sylvester et al. (2024).

Information gained from the development of the RCs, as used in the above studies at UoWBL, include how to best achieve statistically uniform RF‐EMF exposure throughout the RC volume in which the mice are placed, and how to accurately characterize the exposure including the variation in thermal response in the mice. With RCs becoming the default choice for large‐scale exposure systems for mice (and rodents generally), such information is essential for a full and proper characterization of the dosimetry. It is also essential that a good understanding of the thermal response is obtained when seeking to determine if non‐thermal biological effects are caused due to RF‐EMF.

In this paper, we present the relationship between the RF power delivered to the UoWBL chambers and the WBA‐SAR in exposed mice used in the AD study. We also provide a detailed analysis of the uncertainty associated with the WBA‐SAR estimation. Throughout this paper, it is assumed that the frequency of exposure is 1950 MHz (as per the Korean studies). A total of 32 female mice will be undergoing sham or actual exposure. The age of the female mice spans from 6 to 40 weeks with an average mass ranging from around 18.2−27.8 g over this time period and the overall average mass over the time period being around 23.6 g. These values are from measurements taken at UoWBL and from The Jackson Laboratory (2024). The phrase “phantom mice” refers to plastic tubes (with electrical conductivity assumed to be *σ* = 0 S/m, and relative permittivity *ε*_*r*_ = 3) containing tissue simulant liquid (with properties *σ* = 1.4 S/m, *ε*_*r*_ = 40, as used in Gong et al. [2017] for mice), provided by EMC Technologies, Keilor Park, Australia (consisting of surfactant, water, and bactericide). The phrase “phantom model” refers to a computational model of phantom mice. The phrase “mice model” or similar, refers to heterogeneous multi‐tissue computational models of mice. The symbols “< >” are used when denoting an average value (a fuller explanation is provided below). No live animals were used for the development of the RCs. The word “rodent” is used when discussing both mice and rats.

## 2 Literature Review

### 2.1 Dosimetry in AD Studies

In the studies performed in Korea that investigated cognitive and AD pathology outcomes in mice, the RF‐EMF exposure of the mice was performed in an RC of dimensions 2.3 m × 2.3 m × 1.5 m, under controlled environmental conditions, with discussion on the dosimetry referencing Lee et al. (2012). Whilst Lee et al. solely consider dosimetry with rats (for experiments considering a different biological endpoint), Son et al. (2016) indicate that the dosimetry for the Korean AD studies used a computational mouse model consisting of 40 tissues and a voxel length of 1 mm. An important consideration in the Korean AD studies was the thermal response to the exposure. In Jeong et al. (2015), Son et al. (2016), and Jeong et al. (2020), they reported that rectal temperatures were measured before and immediately after exposure and that the temperature changes ranged from −1.9 to +0.5°C, and the authors asserted that the body temperature was thus maintained within the normal range.

### 2.2 The Use of RC as Exposure Systems for Animal Studies

In recent years, the use of RCs appears to have become the preferred exposure environment for whole‐body in vivo experiments. Previously, Chou and Guy (1982) and Chou et al. (1985) showed the benefit of constraining rodents to specific locations within a waveguide or a small anechoic chamber so that energy absorption in the rodents could be readily and precisely characterized. This was also the case with the use of a Ferris Wheel (radial cavity) exposure system as described in Tay et al. (2000), Faraone et al. (2006), and McIntosh et al. (2010), where mice were placed in individual tubes. However, a concern with rodents being constrained is that this may increase stress levels, which may, in turn, confound the assessment of the effects of RF‐EMF on biological endpoints.

In RCs, animal subjects are allowed to roam unconstrained within their non‐metallic cages, and coupled with suitable environmental conditions, this freedom of movement has the likely benefit of reducing stress levels. Kim et al. (2022) also found that the thermophysiological response by rats to RF‐EMF is improved when rats are free‐roaming. However, such movement has the disadvantage that proper dosimetry requires the consideration of the changing orientations of the rodents and the incident EMF, as well as differences in absorption when rodents are isolated compared to when they congregate in groups.

To aid proper characterization of energy absorption in free‐moving animals, the field in an RC is forced to continuously change by using “mode” stirring techniques that ideally create a statistically uniform field environment in a defined volume of space (the working volume; WV) in which all directions of arrivals of the field are equally probable and the magnitude of any field component (e.g. |*E*_*x*_|) is Rayleigh distributed as described by Hill (1998). In a practical RC the WV will contain stirred energy (Rayleigh distributed), and unstirred energy related to fields in the WV that arrive directly from the transmitting antennae. The field in the chamber can be described by the Rician distribution and the *K*‐factor of the distribution, the ratio of the unstirred to stirred energy, is a measure of how well the field in the RC approximates a Rayleigh distribution (*K* = 0). Values of *K* < 1 indicate that the RC environment is predominantly Rayleigh distributed which is the RC design requirement in many applications, such as in animal studies.

Different types of stirring mechanisms can be used to create field uniformity within the WV. The study by Serra et al. (2017) describes the benefits and challenges of some of the key approaches taken to RC design. A popular and well‐studied approach, first developed for use in electromagnetic compatibility testing, is “mechanical stirring,” whereby a large metallic scatterer is placed within the RC and rotates slowly, altering the field structure by stirring the field emanating from a single source antenna positioned to direct its radiated energy toward the scatterer (while simultaneously minimizing direct energy toward the object under test/study). This is the approach taken in the US National Toxicology Program (NTP) study on the health effects of RF‐EMF in wild‐type rats and mice (the findings are available at NTP [2018a, 2018b]). The dosimetry is described in Capstick et al. (2017), Gong et al. (2017), and Wyde et al. (2018).

An alternate RC design, often named “electronic stirring,” is one based on the use of multiple source antennas within the chamber. The use of multiple source antennas has been studied as a way of creating a pattern of excitation that generates a uniform time‐averaged field in the WV (Cerri et al. 2005; Cozza et al. 2012). One such implementation is described in the study by Wu et al. (2010), where six fixed source antennas are used, one on each chamber wall, and a small mechanical mode stirrer positioned in each of the three top corners of the chamber to enhance stirring of the field. Under software control, only one antenna receives RF power at any moment in time, with power cycled through each antenna at the rate of one antenna per second and one rotation of the stirrer per second.

To create computational simulations of exposure that simulated a Rayleigh environment in an RC, Capstick et al. (2017) used a “simplified 12‐plane wave model for the average exposure in a reverberation chamber”—the 12‐plane waves being composed of six waves spaced at 90° and each at two polarizations. Gong et al. (2017) found that the difference in computed exposure between the use of 12‐plane waves compared to a larger set of multiple‐plane waves with random amplitude, phase, polarization, and incident angle was 1.8% for mice and 2.4% for rats. Ito et al. (2022) performed a comprehensive evaluation, including determining when convergence occurs using multiple plane waves of the same amplitude and phase incident on a single rat (WBA‐SAR values were within 1% when using 30 or more), and comparison with plane waves of varying phase and amplitudes that satisfy a Rayleigh distribution in accordance with RC theory (finding an uncertainty no greater than ±5%). They found the preferred model consisted of 52 plane waves of the same phase and this exposure model was utilized for the work described in our paper.

### 2.3 SAR and Thermal Considerations in Rodents

In Chou and Guy (1982), the authors exposed mice in a waveguide and also a small anechoic chamber, subject to 2450 MHz RF‐EMF. The temperature and humidity were kept constant through ventilation. For a 1 W input, the WBA‐SAR was around 3.6 W/kg. Using a hairless mouse, thermography and calorimetry methods were performed to estimate SAR levels. When the mice were facing the center of the waveguide, the maximum SAR was in the head of the mouse. However, when the mice were in the anechoic chamber, the maximum SAR was at the base of the tail, both when the mice were parallel and perpendicular to the incident *E*‐field. The authors noted that the incident current density was higher in the small cross‐sectional area of the tail than in the larger cross‐sectional area of the body.

Chou et al. (1985) conducted measurements on rats, using the same exposure systems as above as well as in a large RF‐shielded room, with the rats subject to various exposure conditions. It was found that “Intensive coupling of energy to the tail when it was exposed parallel to the *E*‐field was shown by thermography.” The paper also found significant SAR levels in the hypothalamus under certain exposure conditions. They noted that both the tail and the hypothalamus are known to be involved in thermoregulation in rats, so studies involving rats need to be aware that observed biological effects may be related to thermoregulation. With such localized thermal effects, they concluded that WBA‐SAR may not be a predictor of the biological effects of microwave exposure.

Ijima et al. (2023) exposed rats with a 28 GHz lens antenna at WBA‐SAR levels 3.7 and 7.2 W/kg, finding that heat generated in the torso was dissipated via increased blood perfusion to the tail. This study emphasized the role of the rat's tail in thermoregulation, as originally observed in papers that they referenced, including Tsuchiya (2001).

Thermoregulatory stress was also examined by Kuhne et al. (2020), who had concerns of its role in the findings of carcinogenicity in rats in the NTP study (NTP 2018a, 2018b), especially at WBA‐SAR levels of 6 W/kg and higher. However, thermoregulatory stress was less likely to affect mice at these exposure levels which, due to their smaller size (and thus greater surface area to volume ratio), have a proportionally greater capacity to lose heat to the environment.

Jeon et al. (2022) described videoing of rats over a 22 h period to assess their changing posture with time and used that information to quantify the influence each position had in the determination of SAR. Similarly, information about the changing posture of mice with time was obtained and used as input into the computational analysis for determining uncertainties in the estimation of WBA‐SAR.

## 3 Design, Build, and Performance of the RC

### 3.1 Design and Build

The design of the UoWBL RCs (operating at 1950 MHz) is based on the RC described in Wu et al. (2010) with chamber dimensions 1.5 m × 1.5 m × 1.5 m, which were constructed using metal panels and built to facilitate exposures in the frequency range from around 860−2600 GHz. The lowest useable frequency for a chamber of these dimensions was determined by Li et al. (2016) to be 750 MHz. The chambers use a form of electronic stirring (Serra et al. 2017), employing six antennas, one on each wall of the chamber, and RF excited in turn to create a statistically uniform and statistically isotropic field in the WV; as well as mechanical stirring using three small stirrers.

The dimensions, metallic construction, and frequency range meet the basic requirements for an RC outlined by Hill (1998) that it be an electrically large, multimode cavity with a high Quality Factor (*Q*) that creates a statistically uniform (time‐averaged) field within the WV by employing various methods of “mode” stirring (Holloway et al. 2006; Wu 2009; Wu et al. 2010; Capstick et al. 2017).

**Figure 1:**
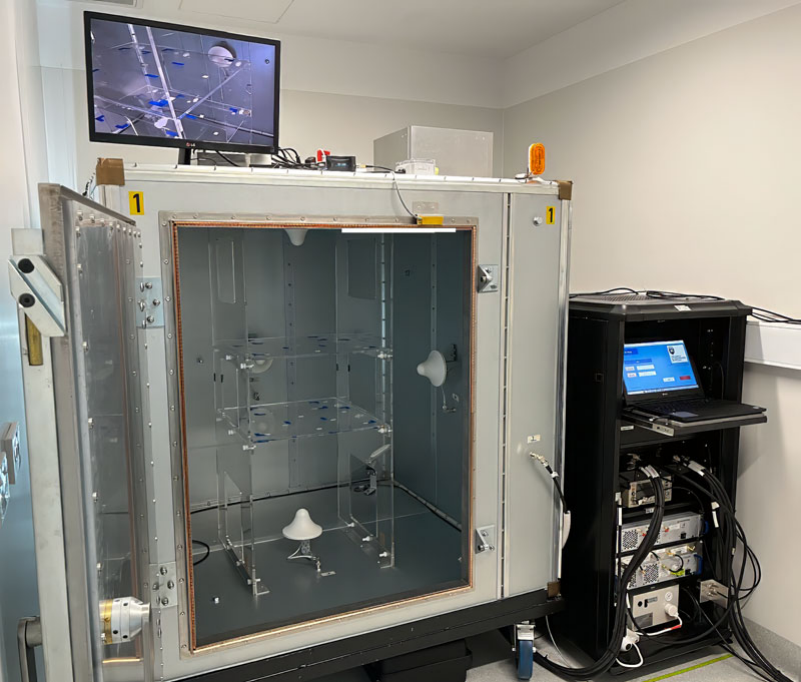
One of the RCs at UoWBL shows top and bottom shelving within the RC and associated equipment rack. Also visible are four of the six antennas, one of the three stirrers (bottom back right), with a video monitor placed on top of the RC. An antenna is placed approximately centrally on each of the sides of the RC, except for the front, where an antenna is placed on the panel to the right of the door.

Two RCs were built for UoWBL, both identical in design and capable of being used for either actual or sham exposure as part of the blinding protocol; 1.45 m × 1.45 m × 1.45 m in internal chamber dimensions (manufactured by Compliance Engineering, Keysborough, Australia) (see Figure 1). The chambers are modular in design, assembled from prefabricated metal alloy plated sheets (55% aluminum and 45% zinc, as commonly manufactured in Australia) laminated to timber panes with steel framing joints providing RF integrity at the panel intersections. Each chamber includes a 1.2 m × 0.9 m shielded door, a 0.5 m × 0.5 m shielded mesh window, two 0.3 m × 0.3 m steel hex honeycomb air vents, a mains RF power filter, and a penetration panel with N‐type bulkhead connectors.

A statistically uniform field within the WV of the chamber (measuring approximately 50 cm × 50 cm × 40 cm = 0.1 m^3^), was achieved using six small profile antennas (one per RC wall) with radiation patterns that are skewed to their sides, directing energy toward the RC walls rather than toward the center of the RC. Small mechanical mode stirrers were positioned in each of the three bottom corners of the chamber to enhance stirring of the field. The metallic stirrers have blades of around 30 cm in total length and 6 cm width. The base of each stirrer consisted of rubber padding and, combined with the slow rotation speed, helped to minimize noise and vibration. The computer control software was developed to enable RF power to be directed to each of the six antennas sequentially via RF electromechanical switches (RF‐SWs). Each antenna was active for a period of 10 s including a 0.5 s minimum power guard period during switching. Mechanical stirrers operated at around 0.5 turns per second (30 revolutions per minute [RPM]). The time required to cycle through a set of six antennas is 1 min. The cycle time was chosen to be shorter than the time constant (time taken to reach 63.2% of the final, steady state value) of a mouse's whole body thermal response. Modeling of a single 23.6 g mid‐sized female mouse showed that the time constant is of the order of 2−4 min (these values were calculated using the mouse models as described below and with the thermal modeling capabilities as used in McIntosh et al. [2010]).

A shelving system was purpose‐built using the material perspex (clear and 10 mm thick), with the design of the system aimed at enabling suitable airflow for the mice and limiting the RF‐EMF scattering and absorption in the shelving. Two horizontal shelves stacked vertically 30 cm apart were placed within the WV of each RC. Four cages (Tecniplast, Lane Cove, Australia; 1144B‐HT), made from polysulfone, can be placed on each of the shelves. A typical setup for the mice is where four cages are placed on each of the two shelves, with four mice per cage.

Within each RC, ancillary equipment includes RF‐EMF and air temperature monitoring and a video camera (with RF shielding to provide immunity to *E*‐fields in the chamber) to monitor the animals, if required. A low‐noise air extraction fan, attached with a rubber mat base to a fine metallic mesh panel on the top of each RC, provides suitable airflow for the mice, with the air input through a second mesh panel inserted into one of the sides of each RC. Air in the chambers was fully refreshed every 2 min. Given the expected limited duration of the experiments per day of between 1 and 3 h, no provision was made for the dispensing of food or water for the mice. A half tissue was included in each cage that served as nesting material as well as to absorb urine. As this addition was minimal, it was not accounted for in our analyses.

**Figure 2:**
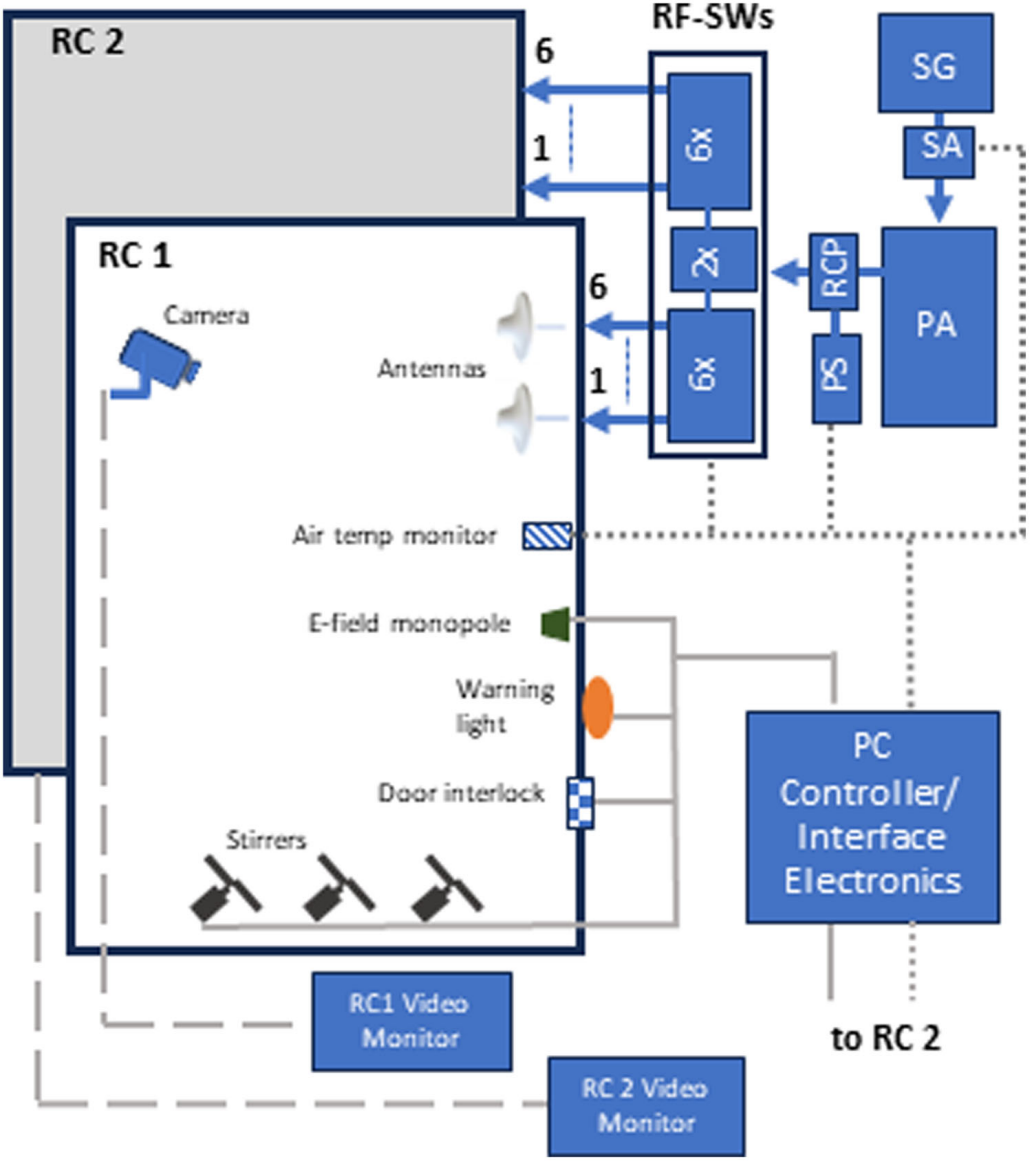
Schematic diagram of the UoWBL reverberation chamber (RC) exposure system showing key elements of the monitoring and control system. Signal cables: USB (dotted line) and analog signals (dashed and solid line).

A block diagram of the UoWBL exposure system showing key elements of the monitoring and control system is given in Figure 2. The RF‐EMF into each RC is provided by a signal generator (SG) (Tektronix TSG 4106A), a digital step attenuator (SA) (Mini‐Circuits, New York, USA; RCDAT‐6000‐60), an RF power amplifier (PA) (Exodus Advanced Communications, Las Vegas, USA; AMP2004), a power sensor (PS) (Mini‐Circuits; PWR‐6GHS) attached to an RF coupler (RCP) (leader‐mw LDDC‐1/6‐40N), and RF‐SWs (JML, Guangdong, China; JR760S062114) feeding the signal into the antennas (Rojone, Ingleburn, Australia; AI‐467‐O‐30‐NF, 698‐2700 MHz).

A laptop PC is employed as the system controller. With software written specifically for this project, the PC controls RF power levels, cycling the power to each of the six antennas, and the operation of ancillary equipment, via the purpose‐built Interface Electronics, including turning the stirrers ON/OFF, monitoring of door sensors (that provide OPEN/CLOSED status of shielded doors) and warning lights indicating that the system is in operation. It also monitors air temperature using a temperature sensor that samples air being extracted from the RC by the air extraction fan. An electrically short *E*‐field monopole mounted near a wall in each RC monitors the presence of a field. The RCP/PS at the output of the PA and the *E*‐field monopole in the chamber verifies that there is continuity in the flow of RF power from the RF amplifier through the RF switches and into the RCs. The software maintains RF power at predetermined levels into the RC through a continuous cycle of measurement via the RF PS and appropriate adjustment of the SA that takes account of RF losses in the coaxial cables and through the RF switches. RF losses were determined through a process of calibration. The relationship between the RF power into the chamber and WBA‐SAR is discussed later in this paper.

As well as controlling exposure levels in the chambers, software was developed to facilitate the double‐blinding of studies. This includes not displaying which of the two RCs has RF exposure (the lights indicating RF is on are illuminated for both chambers regardless of which chamber may be being used for sham exposure). The software guides a researcher through steps necessary to setup the exposure each day. Power levels are determined after the researcher enters the total mass of the mice (see discussion to follow on the power calculations), with the experimenter not knowing which is the exposed or control group. The software then specifies in which of the two RCs each group of mice is to be placed. The ancillary equipment is powered in both.

### 3.2 Performance

Important performance metrics for an RC used for animal exposure are the chamber quality factor *Q* and Rician *K*‐factor (a measure of the chamber's “stirring” efficiency), *E*‐field uniformity and isotropy, and WBA‐SAR efficiency and uniformity in the exposed animals (Capstick et al. 2017). In particular, the Rician *K*‐factor and the *E*‐field uniformity and isotropy are important criteria to allow simplified modeling of energy absorption in exposed animals based on an assumed Rayleigh distribution (fully stirred chamber). WBA‐SAR efficiency is discussed in the section WBA‐SAR ESTIMATION AND VALIDATION and uniformity is discussed in the section UNCERTAINTIES IN THE ESTIMATION OF WBA‐SAR.

The quality factor *Q* of an RC is simply a figure of merit that describes its ability to store energy. It characterizes the resonance of the cavity and determines the mean *E*‐field strength for a given input power (Xu et al. 2021) and can be defined as the ratio of the power stored in the cavity (volume) of the chamber to the power dissipated in its walls and other objects in the chamber. For an RC to perform as intended, Holloway et al. (2006) states that the *Q* must exceed a threshold value *Q*_*thr*_ given by:

(1) $$Q_{\mathrm{thr}}={\left(\frac{4\pi }{3}\right)}^{2/3}\frac{{V_{\mathrm{ch}}}^{1/3}}{2\lambda }$$

where *V*_*ch*_ is the volume of the chamber and *λ* the wavelength at a frequency of 1950 MHz and a chamber volume of 1.45^3^ m^3^, *Q*_*thr*_ ≈12.

The expression for the *Q* of an RC is given by Andrieu et al. (2019):

(2) $$Q=\frac{\omega \varepsilon V_{\mathrm{ch}}<{\left|E_{T}\right|}^{2}>}{P_{T}}$$

where *P*_*T*_ is the input power to the chamber, |*E*_*T*_|^*2*^ the total *E*‐field strength, and the symbols < > representing averaging over stirrer positions. In this paper, < > will extend to averaging over multiple measurement/computational values where stated. For assessing the *Q* of the unloaded (empty) UoWBL RCs, <|*E*_*T*_|^*2*^> includes averaging of 39 *E*‐field strength measurements in the WV, 13 points on each of three horizontal planes consisting of top and bottom shelves (that support non‐metallic laboratory animal cages), and a temporary foam center plane shelf positioned midway between the top and bottom shelves. Planes and measurement points were spaced λ apart (15.4 cm at 1950 MHz) to minimize correlation. At each measurement position, the instantaneous total field |*E*_*T*_| and its three orthogonal components were measured using a calibrated Narda PMM EP600 (NARDA Safety Test Solutions, Italy) three‐axis isotropic electric field probe (100 kHz–9.25 GHz) with fiber optic connecting cable. The maximum dimension of the EP600 probe, tip to tip, is 53 mm. The computational modeling included averaging of the field to account for the finite size of the probe. Measurements were taken every 0.5 s over a 6 min period (720 samples of the field).

Applying Equation 2, the *Q* for the unloaded chamber of volume 1.0^3^ m^3^ (reduced from 1.45^3^ m^3^ due to the forward projection of the six wall‐mounted antennas and the corner positioned mode stirrers) was estimated to be 550, which is 45 times greater than the threshold value *Q*_*thr*_. Additionally, *Q* was determined for a chamber loaded with 32 phantom mice, each tube (92.5 mm length × 25 mm diameter) containing tissue simulant liquid of mass 23.6 g (similar to a mid‐sized female mouse) giving a total mass in the chamber of 0.755 kg. The phantoms were positioned on the two shelves with 16 per shelf. A set of 13 *E*‐field strength measurements were performed at points spaced at least *λ*/2 apart on the center plane located midway between the shelves. Based on these measurements, the *Q* was estimated to be 200, which easily exceeds *Q*_*thr*_ as required. As a point of comparison, a conservative calculation based on a chamber volume equal to the WV gives *Q* values of 55 and 20 for the unloaded and loaded chamber, both values exceeding the threshold value *Q*_*thr*_.

Statistical field uniformity and isotropy were determined in an unloaded and loaded chamber using the measurement data obtained during the assessment of quality factor *Q*. The standard uncertainty in field uniformity and isotropy for an unloaded chamber was 0.96 dB and 1 dB, respectively, at 1950 MHz. In a loaded chamber with 32 phantoms spread across the top (16) and bottom (16) shelves, measurements were performed at 13 points on the foam center plane, midway between the top and bottom shelves. The standard uncertainty in field uniformity and isotropy was 0.92 and 1.2 dB, respectively. In comparison, the standard uncertainty in the uniformity and isotropy of the unloaded NTP chambers was 0.48 and 0.70 dB, respectively, at 1900 MHz. For NTP chambers loaded with animals at 1900 MHz, the standard uncertainty in field uniformity and isotropy was 0.7 and 1.3 dB, respectively. The field uniformity for the chamber described by Wu (2009), in terms of the standard uncertainty of 24 field strength measurements (eight corner positions of the WV and three orthogonal field components), was found to be 1.2 dB at 2450 MHz. No specific reference is made to field isotropy.

**Figure 3:**
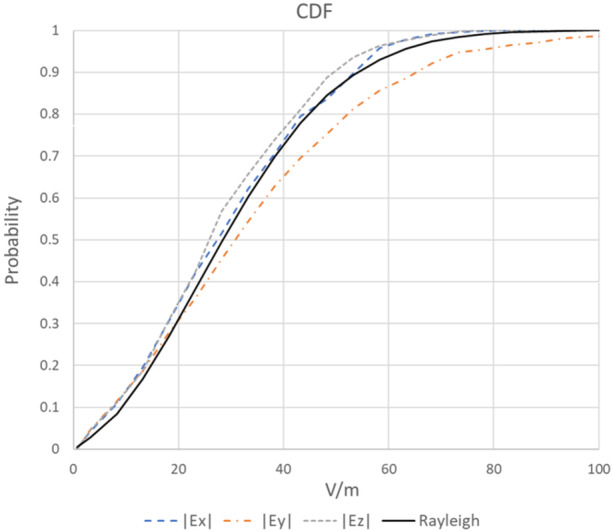
Typical distributions at measurement points. CDFs from one of the 39 measurement points show distributions of individual components of the field and a Rayleigh distribution (*K* = 0) for comparison. Fitting a Rician distribution to the components yields the following *K*‐factors and stirring efficiencies (%): 0.1622 (86%), 0.1682 (85.6%), and 0.2514 (79.9%) for |*E*_*x*_|, |*E*_*y*_|, and |*E*_*z*_|, respectively.

The *K*‐factor was assessed in empty UoWBL chambers and was determined by “fitting” a Rician distribution to each of the cumulative distributions of the measured field components. As an example, cumulative distribution functions of the field components at one of the 39 measurement points and a Rayleigh distribution (*K* = 0) for comparison purposes are shown in Figure 3. The average *K*‐factor over all measurement points is 0.2097 (standard deviation 0.1622) equating to a UoWBL chamber stirring efficiency of 82.7%. In comparison, Capstick et al. (2017) state that the empty NTP chambers achieved a *K*‐factor of 0.003 at 1900 MHz which equates to a stirring efficiency of 99.7%. A beneficial consequence of a highly stirred chamber (e.g., NTP) is that the standard uncertainty in the uniformity of the field will be smaller than that of a relatively less well‐stirred chamber (e.g., UoWBL) which will in turn contribute to a relatively smaller uncertainty budget. Overall, we can conclude that the EMF in the UoWBL chambers is predominantly stirred, and computational modeling based on Rayleigh statistics can be used to predict RF absorption in mice subject to the uncertainty bounds discussed in the section UNCERTAINTIES IN THE ESTIMATION OF WBA‐SAR and summarized in Table 1.

**Table 1:** Uncertainty estimation of WBA‐SAR in mice.

Categories of uncertainty	Component of uncertainty	Dist	Standard uncertainty (dB)
Field probe	Calibration	Norm	0.2
	Isotropy	Rect	0.17
RC performance	*E*‐field strength uniformity	Norm	0.92
	*E*‐field strength isotropy	Norm	1.2
	Stirrer rotations	Rect	0.12
	Power control into RC	Rect	0.14
Tissue simulant	Dielectric properties	Rect	0.11
Computation	Plane versus random waves	Rect	0.08
	WBA‐SAR expression fit	Rect	0.28
	Mice models	Rect	0.04
	Variation in mice postures	Rect	0.13
	WBA‐SAR uniformity	Rect	0.39
Exposure estimation	Hybrid method	Rect	1.06
Combined standard uncertainty (u_c_)			1.94
Expanded uncertainty (2 × u_c_)			3.89

Abbreviations: Dist, distribution; Norm, normal; Rect, rectangular.

## 4 WBA‐SAR Estimation and Validation

### 4.1 Computational Analysis of WBA‐SAR

**Figure 4:**
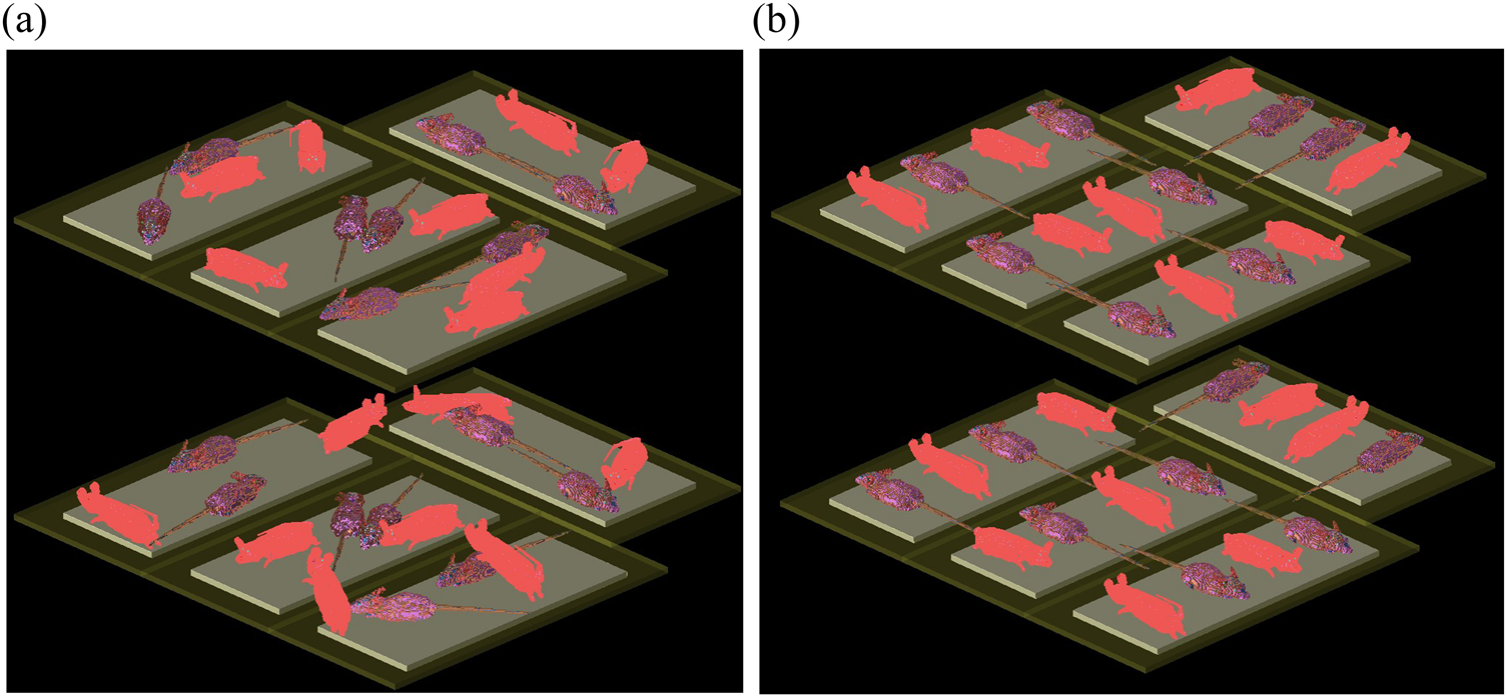
(a and b) Computational models, showing the two shelves of mice with four mice per cage. The different colors of the Swinburne and IT'IS mice are not meaningful and is an artefact of the different input data formats and does not affect the calculations. (a) Shows mice arranged randomly to mimic typical behavior, while (b) shows a uniform arrangement that matches the tubes shown in Figure 5.

**Figure 5:**
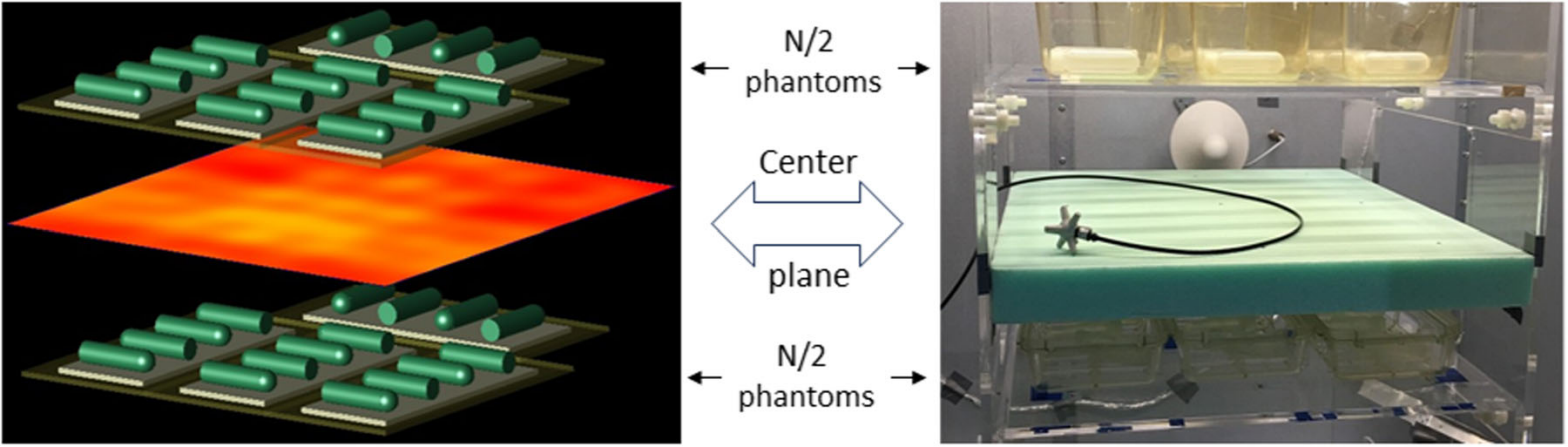
Hybrid method. In the computational model (left side, showing a scenario where there are four phantom mice in each cage), computations were performed to determine the mean square field <|*E*_*mod*_|^*2*^> on the center plane across 13 sample points and the average WBA‐SAR across all *N* phantom mice. In the reverberation chamber (RC; right side), measurements were performed to determine the mean square field <|*E*_*meas*_|^*2*^> across 13 sample points on the center plane for a constant *P*_*in*_ to the RC antennas. The computation (left side) shows the center plane distribution of the magnitude of the *E*‐field with a 1 dB difference between red (the peak) and orange colors. (The data was generated through user‐written code, calculating the average over the 52 plane wave simulations of the square of the magnitude of the *E*‐field at each point. The square root of these values was then fed back into XFDTD to create the plot.).

The electric fields in the RC (*E*, expressed in V/m) and the induced SAR in the mouse models (see Figure 4) and phantom models (see Figure 5), were calculated using the commercially available finite‐difference time‐domain (FDTD) software XFDTD (Remcom 2024; McIntosh et al. 2010). A cubical mesh was used in XFDTD with each cube of side length 0.75 mm. The electrical tissue properties used are from Gabriel (1996).

Modeling the full RC loaded with the mice or phantom models was beyond the capabilities of the computational software and hardware, so an alternate approach using multiple plane waves was employed (as discussed above in the LITERATURE REVIEW section). Each assessment of *E*‐fields in the RC and SAR in the mice consisted of 52 exposure scenarios comprising 26‐plane waves, evenly spaced at 45°, each at two different polarizations, with the plane waves directed toward the center of the model. A typical model of the mice or phantoms positioned on the two shelves took several days to compute using XFDTD, running the 52 exposure scenarios consecutively.

Two female mice models were used in our computations. One of the mice described in McIntosh et al. (2010), which we refer to as the “Swinburne” mouse, consisting of 37 tissues, and was discretized using a 0.75 mm mesh. The second mouse was generously provided by IT'IS Foundation, which we refer to as the “IT'IS” mouse, consisting of 48 tissues, with a 1 mm mesh (which we re‐meshed to 0.75 mm) (IT'IS Foundation 2024). It was important to use two mouse models to add variability into the analyses to reflect real‐life variability. Shape‐wise, the Swinburne mouse is a little more elongated than the IT'IS mouse (see Figure 4). The different shapes are appropriate given that such differences reflect changes as mice move.

### 4.2 Methodology of Determination of Power Into Chamber to Obtain Desired WBA‐SAR Level in Mice

An important consideration in designing the RCs was determining the net power *P*_*in*_ into the chambers that produced a desired WBA‐SAR in mice. The approach adopted in this study is based on the method outlined in Wu et al. (2010) described as a hybrid method (see Figure 5). In brief, an RC is loaded with a predetermined number of phantoms placed on non‐metallic shelves and the spatial field over a center plane (right side of Figure 5) is measured for a given power into the RC (e.g., 1 W). Separately, computational modeling, based on the same number and mass of phantoms in the RC and similarly arranged, is performed to determine the spatial field over a similarly positioned center plane (left side of Figure 5) for a given incident field strength (e.g., 1 V/m) and the consequent WBA‐SAR in the exposed phantoms. The WBA‐SAR for an input power of 1 W into the RC is determined by scaling the computationally derived WBA‐SAR so that the computed field strength on the center plane matches the measured center plane field strength in the RC (*P*_*in*_ = 1 W). Further modeling is performed to adjust phantom‐derived results to actual mice. This method establishes a relationship between the net power *P*_*in*_ into the chambers and desired WBA‐SAR in RF exposed animals.

The first step toward calibrating the RCs was to use phantom mice to determine *P*_*in*_ that produced the desired WBA‐SAR. The results were then extrapolated to actual mice by comparing computational models with phantoms composed of homogeneous tissue simulant liquid and an animal mouse model composed of heterogeneous tissues (using equal numbers of Swinburne and IT'IS mice). Initial computational modeling involved *N* phantoms of 23.6 g (representing the mass of mid‐sized female mice) split into two groups of *N/2*, each group placed on horizontal layers, spaced 30 cm apart vertically (see left side of Figure 5 for the scenario where *N* = 32), simulating the 10 mm perspex shelves. Analysis was performed for three values of *N* (24, 32, and 40) resulting in a total mass in the chamber of 0.57, 0.76, and 0.94 kg, respectively. This mass range ensured that the analysis covered the growth of 32 female mice over the exposure period, from 6 to 40 weeks, from an average mass of 18.2−27.8 g. Additional computational modeling was performed at an average female mouse mass of 18.2 g (average expected mass at 6 weeks) and 27.8 g (40 weeks), and the results fed into the uncertainty analysis as discussed later.

Measurements were made of the *E*‐field strength at 13 points on the center plane in the RC, the points being spaced *λ* apart and at an RF power level into the RC set to *P*_*in*_ = 1 W. This led to the following relationship between *P*_*in*_ and the measured mean squared value <|*E*_*meas*_|^*2*^> over the sample points:

(3) $$P_{\mathrm{in}}=a<{\left|E_{\mathrm{meas}}\right|}^{2}>$$

where *a* is a constant of proportionality.

In the computational model, the relationship between the WBA‐SAR over *N* phantoms, <*WBA‐SAR*>, and the mean squared value <|*E*_*mod*_|^*2*^> of the *E*‐field values over 13 points on the center plane is given by:

(4) $$<\;\text{WBA‐SAR}>=b<|E_{\mathrm{mod}}{|}^{2}\;>$$

where *b* is a constant of proportionality.

The process of equating the mean squared field strength values (Equations 3 and 4) was performed through the least squares method, minimizing the summed difference of the squares of the *E*‐field strength values at the sample points in the computational model and measurement, respectively. The least squares method results in:

(5) $$<\;{\left|E_{\mathrm{meas}}\right|}^{2}>=c<{\left|E_{\mathrm{mod}}\right|}^{2}>$$

where *c* is a constant (representing the scaling factor between model and measurement).

The relationship between *P*_*in*_ and <*WBA‐SAR*> was determined by combining Equations 3, 4, and 5 to obtain the following relationship:

(6) $$<\;\text{WBA‐SAR}>=d\;P_{\mathrm{in}}$$

where *d* is the conversion factor for the RC that includes the ratio *a/b*, and the scaling factor *c*.

Given the various values for total mass *M*, a power fit (similar to Gong et al. [2017]) was applied to the data with the following equation (with the coefficient of determination *R* ^*2*^ = 0.93):

(7) $$<\;\text{WBA‐SAR}>\left(P_{\mathrm{in}}=1W\right)=0.408M^{-0.627}$$

To extrapolate this formula to mice, a second comparison was performed between a computational model with phantoms and a model with mice (equal numbers of Swinburne and IT'IS mice), placed in the same locations, and each with the same mass (see Figures 4 and 5). The average WBA‐SAR for mice was around 93% of the average WBA‐SAR for the phantoms, and an appropriate scaling was applied to the above equation. The SAR sensitivity in the UoWBL chambers, expressed as the WBA‐SAR normalized to 1 V/m incident *E*‐field strength ([W/kg]/[V/m]^2^), is 269 μW/kg/(V/m)^2^ for the 23.6 g female mouse. This value is consistent with NTP value presented by Gong et al. (2017) in Table VI showing that WBA‐SAR sensitivity at 1900 MHz for a 20 g female mouse is 240 μW/kg/(V/m)^2^. The WBA‐SAR efficiency, expressed as the percentage of the power transmitted into the chamber that is coupled into the female mice, is 37.2% for the UoWBL. For the loaded NTP chambers, Capstick et al. (2017) note that the efficiency is ∼45% for adult mice (mix of male/female).

The resultant Equation 7 can then be used by the experimenter, who, after weighing the similarly aged mice, can set the input power to each RC for a desired average WBA‐SAR (which will be 5 W/kg in the replication of the Korean AD study).

### 4.3 Experimental Validation of Computed WBA‐SAR

To provide confidence in the computed estimation of WBA‐SAR, the measurement of temperature based on the “lumped‐heat‐capacity” method was used with homogeneous phantoms (Holman 1997). With this method, the WBA‐SAR in a phantom can be estimated using the formula:

(8) $$\text{WBA‐SAR}\;=c\left(T_{\mathrm{ss}}-T_{i}\right)/\tau$$

where *c* is the specific heat capacity of the material (around 3390 J/(kg°C) for the tissue simulant liquid), *T*_*i*_ (°C) is the initial temperature, *T*_*SS*_ (°C) is the final temperature at steady state, *τ* (s) is the time constant, the time it takes for the temperature to rise from *T*_*i*_ to (1−e^−1^).(*Τ*_*SS*_ ‐*T*_*i*_), and the ambient temperature is constant (Ebert 2009).

Measurements were performed in a chamber loaded with 32 phantom mice, 23.6 g of tissue stimulant liquid in each phantom, and with equal numbers of phantoms on each shelf. A series of six temperature measurements were performed, a set of three measurements in one of the 32 phantoms that was placed vertically in the middle of the top shelf (a position and orientation that provided the lowest calculated WBA‐SAR in the phantom), and the other three in a phantom that lay horizontally on a front corner of the top shelf (the highest calculated WBA‐SAR).

**Figure 6:**
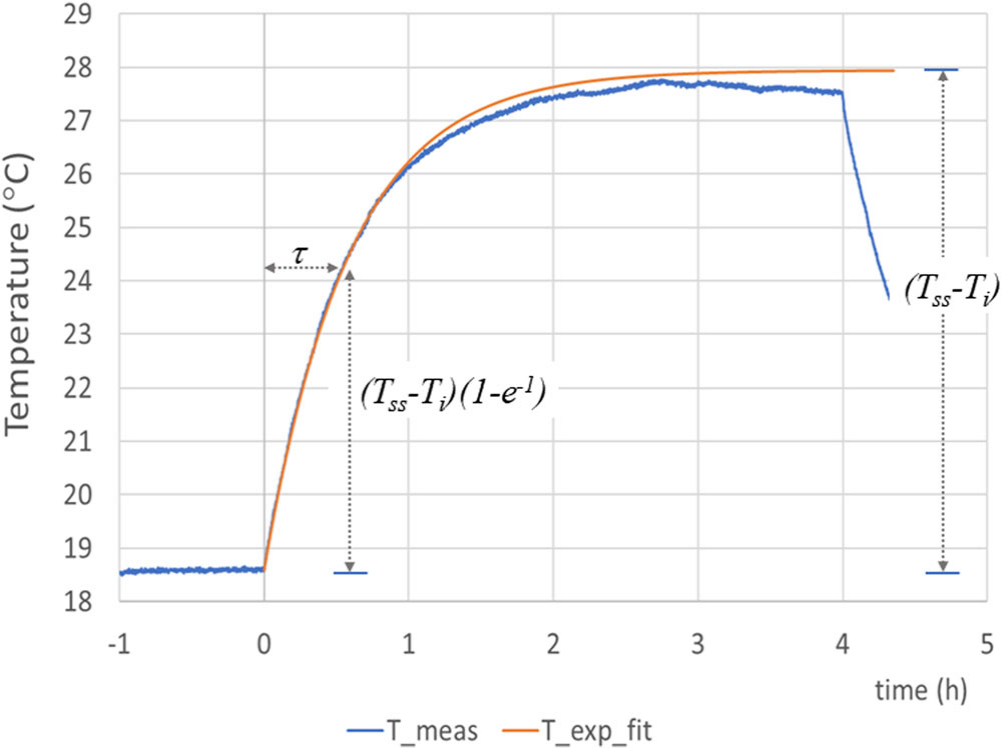
Example of measurement of the temperature rise (T_meas) in a horizontal mouse phantom and the exponential curve fit (T_exp_fit). The total number of phantoms in the reverberation chamber was 32 with a combined total mass of 0.76 kg. The RF was turned ON at 0 h and OFF after 4 h.

The tip of a fluoroptic temperature probe was inserted into the phantom in which temperature was being measured. The probe's associated fiber optic cable was fed through a waveguide opening in a wall of the chamber and connected to a LUXTRON 812 temperature monitor (Luma‐Sense Technologies, Santa Barbara, USA) located outside of the chamber. To satisfy the underlying assumption of uniform temperature throughout the phantom, as required in the lumped‐heat‐capacity method, the phantom was held just above the shelf by nonconductive strings and via the strings, gently rocked from side‐to‐side during exposure to encourage stirring of the simulant liquid. Each of the six measurements were performed over at least a 300 min period, with around 30−60 min pre‐exposure to ensure the temperature within the liquid and the ambient temperature were both equivalent and stable. A steady state was reached after about 3 h of exposure (Figure 6). An important consideration was to minimize changes in ambient room temperature external to the RC during hours‐long exposures since these changes could unintentionally change the temperature in the phantom liquid causing temperature measurements to be compromised. Therefore, external temperature was monitored and recorded and a steady stream of near constant temperature‐controlled airflow was circulated around the RC.

The input power to the antennas was set to 21.88 W, a level chosen to ensure significant thermal effects, so after using Equation 7, the average WBA‐SAR across all phantoms was estimated to be 10.64 W/kg. After comparing the values between phantoms (using a computational model), the WBA‐SAR in the horizontal corner phantom was estimated to be 11.60 W/kg. In comparison, three measurement results evaluated using the “lumped‐heat‐capacity” method provided the values 14.97, 14.28, and 13.09 W/kg for the horizontal phantom.

For the vertical phantom, the computed WBA‐SAR was estimated to be 5.71 W/kg. The reduction in WBA‐SAR for the vertical phantom is primarily due to the different shapes of the liquid within the vertical and horizontal tubes. This occurs because the liquid does not occupy the full volume of the tube. (The reduction was confirmed by separate WBA‐SAR modeling in single vertical and horizontal mouse phantoms taking account of the changing shape of the liquid.) The three measurement results evaluated using the “lumped‐heat‐capacity” method provided the values 5.72, 6.28, and 6.78 W/kg for the vertical phantom.

Across all six measurements, the average ratio of the measured to computed WBA‐SAR was 0.63 dB and the maximum ratio was 1.1 dB, providing confidence that the computational estimates of WBA‐SAR are well matched to exposures in the RCs.

## 5 Uncertainties in the Estimation of WBA‐SAR

Uncertainties in the estimation of WBA‐SAR in the mice arise from numerous inputs. These include the extent to which the field in the WV of the chamber is statistically uniform and consistent with an assumed Raleigh RF‐EMF exposure environment. Additionally, mice will roam freely within the confines of their cages so estimating WBA‐SAR under these conditions is a further input to the assessment of uncertainty. Here we will list the categories and components of uncertainty, stated in terms of a standard uncertainty, and determine the combined standard uncertainty by aggregating all components and stating the result at the 95th percentile confidence interval. Some are estimated using computational modeling while others from measurements (see Table 1).

Starting with computation, we considered the variation of WBA‐SAR in phantom models in the WV as shown in Figure 5. Analysis showed that the variation over 32 phantoms with an average mass of 23.6 g resulted in a standard uncertainty of 0.39 dB. Next, consider the variation of WBA‐SAR across the mice as they change positions. The analysis consisted of comparing the difference in WBA‐SAR for the model when the mice are placed in regimented positions (the same positions of the phantoms as shown in Figure 5) compared to a model with mice in various positions that correspond to typical mice behavior (i.e., a model where mice are placed along the edges of the cages, by themselves, sleeping in a small group, or climbing up the sides of the cages or climbing over other mice, see Figure 4). A total of 32 mice were modeled with an average mass of 23.6 g. When comparing these two scenarios, the WBA‐SAR varied by just 2.88% (0.13 dB). The accuracy of using the power fit equation to estimate WBA‐SAR as a function of total mass in the chamber, as described with Equation 7, is estimated to contribute 0.28 dB. The estimate is based on an analysis of three sizes of mice, 18.2, 23.6, and 27.8 g. As discussed earlier, the use of a set of 52 plane waves to model the fields present in the RC (see the earlier discussion) contributes 0.08 dB to the estimation of total uncertainty in WBA‐SAR (Gong et al. 2017). Gong et al. (2017) also considered the uncertainty in the computational model of rodents (discretization and dielectric properties) and found ±1% (0.04 dB) for mice (1900 MHz).

Now considering uncertainty arising from measurements, the standard uncertainty in the field uniformity and isotropy in the unloaded chamber was found to be 0.96 and 1 dB respectively. The three‐axis probe field strength calibration at 1950 MHz was 0.2 dB and isotropy 0.17 dB. The contribution from a finite number of stirrer rotations in any 1 min cycle time is estimated to contribute 0.12 dB. Control of the RF power level into the chamber was determined to introduce a standard uncertainty of 0.14 dB. The tissue simulant liquid was provided with a documented uncertainty in the dielectric properties of ±2.5% (0.11 dB).

Uncertainty related to the hybrid method, where measurement and computational modeling is combined to determine the WBA‐SAR in mice, was estimated by calculating the root mean square difference between field strength measurements at 13 sample points on the center plane and computed field strengths at the same relative positions (for 23.6 g mice). The standard uncertainty is estimated to be 1.06 dB.

Hence, the expanded total uncertainty (*k *= 2) of the WBA‐SAR is 3.89 dB (Table 1). The size of this uncertainty is consistent with the comprehensive uncertainty analysis by Wu (2009) for an RC, examining variability in computation and measurement in determining WBA‐SAR in rats, which found that the maximum variability (*k* = 2) was up to ±118.3% (3.39 dB). In the US NTP study, Capstick et al. (2017) notes that among “all the models, the uniformity of whole‐body averaged SAR (wbSAR) has the maximum expanded uncertainties (*k* = 2) of 2.9 dB at 900 MHz for small female rats and 2.6 dB for small male mice at 1900 MHz.” The Korean studies were based on the RC design by Lee et al. (2012), in which the authors state that measurements using a 3‐axis isotropic probe on the surface of the polycarbonate cage holding the animals found that “The field distribution of both 848.5 and 1950 MHz was well within 3 dB in the region of interest.” No further exposure uncertainty analysis is provided. We can compare uncertainty estimates for RCs with that of the animal constrained in the 900 MHz Ferris‐wheel (radial cavity) exposure system described in Faraone et al. (2006), who states that, when using mouse cadavers as test subjects, “Over the selected range of weights and locations, the peak‐to‐peak normalized SAR variation is about ±1.1 dB.” Validation of WBA‐SAR estimates based on temperature measurements in phantom mice found that the maximum ratio of the temperature‐derived WBA‐SAR to the computed WBA‐SAR was 1.1 dB, suggesting that actual exposures are likely to be well within the expanded uncertainty shown in Table 1.

## 6 Conclusions

In this paper, we have presented the design, RF‐EMF performance, and a comprehensive uncertainty analysis of the RC exposure system that has been developed for the use of researchers at UoWBL for the purpose of investigating the biological effects of RF‐EMF, with initial application for mice. An RC allows relatively unconstrained movement of rodents during exposures compared to physical restraints imposed on animals in other exposure systems such as in TEM cells and radial waveguide systems. This leads to a key benefit of RCs in that by allowing animals to roam they can minimize the potential stress caused by constraining the animals during RF‐EMF exposure which in turn can minimize experimental confounders related to non‐RF‐induced biological and behavioral changes in the animals.

The exposure system was calibrated, and control software was developed to ensure that RF input power into the chambers was adjusted to keep the WBA‐SAR dose constant using the actual mass of the animals over their life span. As well as field strength measurements and computational analysis, temperature measurements based on the “lumped‐heat‐capacity” method were performed using mice phantoms to validate the RF input power levels. The control software enabled real‐time monitoring of the state of exposure during experiments and facilitated double‐blinding. An RF‐shielded video camera system installed in each chamber allows for the monitoring of animals during exposures, if required. Based on a comprehensive analysis of the main sources of uncertainty, the expanded uncertainty (*k* = 2) of the WBA‐SAR is 3.89 dB at 1950 MHz. This is comparable to the uncertainty for exposure reported by Wu (2009) at 2450 MHz but higher than for the NTP study in Capstick et al. (2017) at 1900 MHz. Validation of WBA‐SAR estimates based on a selected number of temperature measurements in phantom mice found that the maximum ratio of the temperature‐derived WBA‐SAR to the computed WBA‐SAR was 1.1 dB, suggesting that actual WBA‐SAR is likely to be well within the expanded uncertainty. In conclusion, the development of RCs for UoWBL has enabled us to gain much understanding of the performance of such chambers including the uncertainties involved in the stating of a given dose. Such understanding is important when evaluating whether a biological effect may be caused by excess temperatures. Further analysis, based on the approach in this paper, will be applied to rats in support of future studies.

## Conflicts of Interest

The first, third, and fourth listed authors are former employees of a telecommunications company; the second author is a current employee of a telecommunications company; the third author is a former employee of a national telecommunications industry association.
